# The impact of hormones on lung development and function: an overlooked aspect to consider from early childhood

**DOI:** 10.3389/fendo.2024.1425149

**Published:** 2024-09-20

**Authors:** Gloria Pelizzo, Valeria Calcaterra, Paola Baldassarre, Michela Marinaro, Silvia Taranto, Michele Ceresola, Gerson Capelo, Cassandra Gazzola, Gianvincenzo Zuccotti

**Affiliations:** ^1^ Pediatric Surgery Department, Buzzi Children’s Hospital, Milan, Italy; ^2^ Department of Biomedical and Clinical Science, University of Milan, Milan, Italy; ^3^ Pediatrics and Adolescentology Unit, Department of Internal Medicine, University of Pavia, Pavia, Italy; ^4^ Pediatric Department, Buzzi Children’s Hospital, Milan, Italy

**Keywords:** lung development, lung function, hormones, lung growth, lung disorders, children, sex hormones

## Abstract

The impact of hormones on the respiratory system constitutes a multifaceted and intricate facet of human biology. We propose a comprehensive review of recent advancements in understanding the interactions between hormones and pulmonary development and function, focusing on pediatric populations. We explore how hormones can influence ventilation, perfusion, and pulmonary function, from regulating airway muscle tone to modulating the inflammatory response. Hormones play an important role in the growth and development of lung tissues, influencing them from early stages through infancy, childhood, adolescence, and into adulthood. Glucocorticoids, thyroid hormones, insulin, ghrelin, leptin, glucagon-like peptide 1 (GLP-1), retinoids, cholecalciferol sex steroids, hormones derived from adipose tissue, factors like insulin, granulocyte-macrophage colony-stimulating factor (GM-CSF) and glucagon are key players in modulating respiratory mechanics and inflammation. While ample evidence underscores the impact of hormones on lung development and function, along with sex-related differences in the prevalence of respiratory disorders, further research is needed to clarify their specific roles in these conditions. Further research into the mechanisms underlying hormonal effects is essential for the development of customizing therapeutic approaches for respiratory diseases. Understanding the impact of hormones on lung function could be valuable for developing personalized monitoring approaches in both medical and surgical pediatric settings, in order to improve outcomes and the quality of care for pediatric patients.

## 1 Introduction

Breathing stands as a vital physiological process shaped by myriad factors, ensuring the provision of an adequate oxygen (O2) supply and the effective removal of carbon dioxide (CO2), thus preserving the delicate acid-base equilibrium within blood and living tissues. The orchestration of respiratory dynamics entails a finely tuned interplay of multiple elements, responsive to the body’s requirements through the modulation of three primary components: a central medullary rhythm/pontine pattern generator and integrator, sensory inputs integrated within the unit, and motor outputs directed toward the respiratory musculature (1, 2).

In recent years, growing research has revealed the important influence of hormonal signaling on lung function, from embryonic development to aging. As key regulators of various cellular and metabolic pathways, hormones exert complex effects on the respiratory system, ranging from modulation of smooth muscle tone to regulation of inflammatory response. While the influence of sex hormones on lung physiology has received considerable attention, recent studies have expanded our understanding to include adipose-derived hormones, cytokines, and other hormones (3, 4).

The impact of sex hormones on the respiratory system constitutes a multifaceted and intricate facet of human biology. The scientific world is witnessing a burgeoning exploration into the nuanced interplay between sex and respiratory physiology, albeit hindered by the limited scope of available research, predominantly focused on male cohorts and attributes. Nevertheless, evidence underscores the pivotal roles played by sex hormones—such as estrogen, progesterone, and testosterone—across various dimensions of respiratory function throughout the human lifespan (5). Chemoreceptors, specialized cells adept at discerning alterations in the partial pressures of CO2 and O2, as well as shifts in pH levels, are ubiquitously distributed across various loci within the central nervous system and additionally found peripherally within the carotid and aortic regions. In both areas, the presence of receptors for sex hormones has been identified, showcasing their genomic and non-genomic influences and affirming their involvement in the regulation of breathing (6, 7). These hormones exert influence ranging from shaping respiratory drive and muscle performance to modulating airway reactivity and lung maturation. Beyond sex steroids, adipose tissue-derived hormones like leptin and adiponectin contribute to lung disease, especially asthma development and severity. Hormonal influence extends to factors like insulin, granulocyte-macrophage colony-stimulating factor (GM-CSF), and glucagon, which modulate respiratory mechanics and inflammation. Hormones play a role in respiratory function even during fetal development, when maternal hormonal environments significantly influence lung morphogenesis and development. The maternal environment during pregnancy can affect the respiratory and immune health of offspring, potentially influencing their vulnerability to respiratory infections in later life. Steroid signaling has been specifically linked to respiratory and developmental outcomes, which are closely related to early life respiratory infections. However, research on the relationship between endogenous steroid levels during pregnancy and offspring respiratory and immune health has shown inconsistent results. While prenatal androgens support fetal lung branching morphogenesis, elevated levels might hinder the offspring’s immune response to pathogens after birth. Similarly, corticosteroids, when administered in late pregnancy, aid lung maturation but also increase the risk of respiratory diseases, such as asthma, in the offspring. Given the close association between steroid levels during pregnancy and offspring respiratory and immune health, steroid metabolites may be relevant to the propensity for childhood respiratory infections and could serve as potential therapeutic targets (8–10).

We propose a comprehensive review of recent advancements in understanding the interactions between hormones and pulmonary development and function, focusing on pediatric populations. We explore how hormones can influence ventilation, perfusion, and pulmonary function, from regulating airway muscle tone to modulating the inflammatory response. Understanding the impact of hormones on lung function could be valuable for developing personalized monitoring approaches in both medical and surgical pediatric settings, in order to improve outcomes and the quality of care for pediatric patients.

## 2 Methods

**Figure 1:**
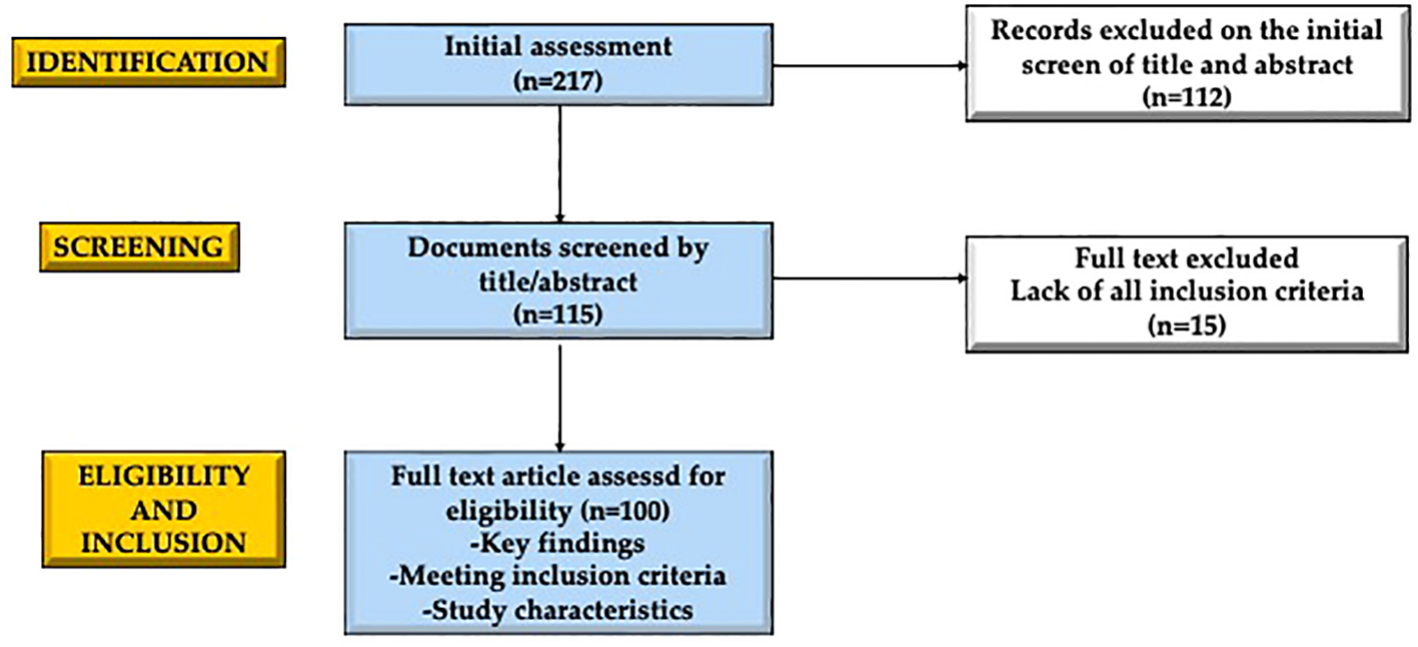
Graphical representation of the manuscript selection and exclusion process employed in crafting this narrative review. The initial assessment included 217 results, from which 112 articles were excluded by evaluating their title and abstract. Of the 115 selected articles, 15 were excluded by reading the entire text. The 100 selected articles respected the key points, the inclusion criteria and the characteristics of the study. Created with BioRender.com ^®^.

We conducted a narrative review to investigate the impact of hormones on lung development and function. Our search included a thorough examination of literature available on the PubMed database and Scopus focusing on English language publications from the past 20 years. Review, original article and randomized controlled trials which involve both adult and pediatric populations were considered. Case reports or series, editorial and letters were excluded. Paper were searched with the following keywords (alone or in combination): lung development, fetal lung, pulmonary function tests, lung function, hormones, lung growth, lung disorders, lung diseases, children, sex hormones. Initially, we began with a pool of 217 articles, which we then refined by screening abstracts (n=115) and subsequently conducting a comprehensive analysis of the full texts of relevant papers (n=100). In **Figure 1**, the process of paper selection and exclusion was resumed.

## 3 Lung development

The respiratory diverticulum grows from the ventral wall of the foregut when the embryo reaches 4 weeks old and measures 3.5 mm. The development of the lung bud depends on an increase in retinoic acid (RA) produced by adjacent mesoderm: as the level of RA increases, the transcription factor TBX4 is expressed in the endoderm of the gut tube at the location of the respiratory diverticulum; TBX4 triggers the formation of the lung bud and facilitates its continued growth and differentiation (11, 12).

Epithelium of the internal lining of the larynx, trachea, bronchi and lungs, is entirely of endodermal origin. Instead, the cartilaginous, muscular, and connective tissue components of the trachea and lungs derive from splanchnic mesoderm surrounding the foregut (13–15).

At first, the lung bud is in communication with the foregut. When the diverticulum develops caudally, two longitudinal tracheoesophageal ridges form, separating the lung bud from the foregut. The tracheoesophageal septum forms from the fusion of the ridges and divides the foregut into the esophagus, the dorsal portion, and the trachea and two lateral bronchial buds, the ventral portion. The respiratory primordium and the pharynx remain connected through the laryngeal orifice.

In the fifth week, the two bronchial buds grow to form left and right main bronchi. The right main bronchus is wider than the left one and has a vertical orientation. Three secondary bronchi originate from the right one and two from the left one, foreshadowing the three lobes of the lung on the right side and two on the left side.

The lung buds expand into the body cavity growing laterally and caudally. The pericardioperitoneal canals are narrow spaces, which lie laterally to the foregut and are gradually filled by the lung buds. Ultimately, the pericardioperitoneal canals are separated from the peritoneal and pericardial cavities by the pleuroperitoneal and pleuropericardial folds. This separation results in the formation of the primitive pleural cavities. The visceral pleura will develop from the mesoderm covering the lungs. The parietal pleura comes from the somatic mesoderm which covers the inside of the body wall. The space between the visceral and parietal pleura is the pleural cavity (16–18).

Around the 7th week, as the lungs develop further, 10 tertiary bronchi on the right side and 8 on the left side originate from the dichotomous division of the secondary bronchi. Each tertiary bronchi, with the mesenchymal tissue that encircles them, will form the first model of the bronchopulmonary segments. The pulmonary artery branches originate during the 20th week. By the end of the sixth month, approximately 17 generations of subdivisions have been formed. However, after birth, an additional six divisions form, which help in shaping the bronchial tree’s final structure. This phase is regulated by epithelial-mesenchymal interactions between the endoderm of the lung buds and splanchnic mesoderm that encircles them. The mesoderm releases signals, consisting of members of the fibroblast growth factor family, that induce branching, and these signals. Cartilaginous plaques, smooth muscle fibers, bronchial and pulmonary connective tissue develop from the mesenchymal ring encircling each tertiary bronchi. As the bronchial tree develops and these new subdivisions occur, the lungs gradually shift to a more caudal position, thus, by the time of birth, the trachea bifurcation is at the level of the fourth thoracic vertebra (19–23).

Up to the seventh month of gestation, the bronchioles continuously divide into smaller canals during the canalicular phase. The terminal bronchioles divide to form respiratory bronchioles, which divide into three to six alveolar ducts. These ducts end in primitive alveoli called terminal sacs, which are surrounded by flat alveolar cells in close proximity to neighboring capillaries.

By the end of the seventh month, there are enough mature alveolar sacs and capillaries to ensure sufficient gas exchange, enabling a premature infant to survive. The number of terminal sacs gradually increases during the last two months of prenatal life and for several years after birth.

The cells lining the sacs, known as type I alveolar epithelial cells, become thinner and the surrounding capillaries protrude into the alveolar sacs. This close contact between endothelial and epithelial cells forms the blood-air barrier. Mature alveoli are not present before birth. Another cell type develops at the end of the sixth month: the type II alveolar epithelial cells. These cells produce a phospholipid-rich fluid that can lower surface tension at the air-alveolar interface known as surfactant. Before birth, the lungs contain fluid with a high chloride concentration, little protein, mucus from the bronchial glands, and surfactant. The amount of surfactant increases significantly, particularly during the last two weeks of gestation (22, 24–29).

During the 34th week of pregnancy, some of this phospholipid enters the amniotic fluid. This triggers macrophages in the amniotic cavity to activate and migrate across the chorion into the uterus. Once in the uterus, the macrophages start producing immune system proteins, including interleukin-1β. Upregulation of these proteins results in increased production of prostaglandins, which leads to uterine contractions. This suggests that the fetus may participate with these signals in initiating labor and birth.

Fetal breathing movements start before birth and result in the aspiration of amniotic fluid. These movements are critical for stimulating lung development and conditioning respiratory muscles. After birth, when the first breaths happen, the majority of the lung fluid is rapidly absorbed by the blood and lymph capillaries, while a small amount is probably expelled during delivery via and bronchi the trachea (30, 31). As the fluid is reabsorbed from the alveolar sacs, surfactant deposits as a thin phospholipid layer on alveolar cell membranes. When air enters the alveoli during the first respiratory act, the surfactant layer prevents the formation of an air-water (blood) interface with high surface tension. Without the fatty surfactant layer, the alveoli would collapse during expiration, resulting in atelectasis.

It is important to underline that the alveolar structures become mature in high percentage (95%), from the neonatal period. After birth, the rhythmical movements of the pulmonary machine promote the progression of the development of the alveolar component of the lung. Most of the end stage of the maturation of the lung happens until the 3rd year of life and keeps developing up until the 8th year of life (32).

## 4 Lung function

### 4.1 Pulmonary function tests in children

The evaluation of pulmonary function in children is a topic of debate. Unlike in adults, the tests used to evaluate pulmonary function in children must be simple and reproducible. Traditional evaluations require cooperation and concentration, which can be difficult for children.

In **Table 1**, are reported the tests usually used to assess pulmonary function, including spirometry, Forced Oscillation Technique (FOT)/Impulse Oscillometry (IOS), Tidal Breathing Flow-Volume Loop (TBFVL), Rapid Thoracoabdominal Compression (RTC), Raised Volume Rapid Thoracoabdominal Compression (RVRTC), Diffusion Capacity of Lungs for Carbon Monoxide (DLCO)/Transfer Factor For CO (TLCO), Lung Volumes (Body Plethysmography), Multiple-Breath Washout Test (Lung Clearance Index) and Exercise Testing (Cardiopulmonary Exercise Test - CPET) (33–36).

**Table 1:** Pulmonary function tests.

Test	Description
**Spirometry**	Measures lung volume and airflow. The value of this test is because it is an assessment-diagnostic, monitoring, and epidemiological tool. The forced vital capacity (FVC) is a 4 steps maneuver. Actually, there’s not a minimum forced expiratory time but is recommended to reach at least 3 seconds in infants and 6 seconds in older children. The forced expiratory volume (FEV) in children under 6 years is in the first 0.75 seconds (33–36). For the interpretation of this test there’s a strong recommendation to use the Global Lung function Initiative (GLI) reference equations, also for DLCO and lung volumes (33). The importance of this study lies in the capacity to differentiate abnormal respiratory function into restrictive, obstructive, or mixed pattern, based on an FEV1/FVC ratio under than 80% of normal values. An improvement of approximately 10-12% of the FEV1 after a bronchodilator (usually Salbutamol) is a sign of reverse of obstructive pattern in spirometry (33).
**Forced Oscillation Technique (FOT)/Impulse Oscillometry (IOS)**	Measures airway resistance during tidal breathing using wave patterns. Suitable for children over 2 years old who cannot cooperate with spirometry (33, 34). Uses parameters like impedance, reactance, and resonant frequency to identify airway pathologies. It is important to note that standardization of reference results for this test is necessary on the basis of anthropometric, clinical and equipment data in order to establish a baseline (33–35)
**Tidal Breathing Flow-Volume Loop (TBFVL), Rapid Thoracoabdominal Compression (RTC), Raised Volume Rapid Thoracoabdominal Compression (RVRTC)**	The Tidal breathing flow-volume loop test involves tidal breathing at rest using a pneumotachograph to obtain different curve patterns. These patterns can be interpreted to diagnose airway obstruction. The normal peak ratio is between 0.3-0.4. This test is advantageous because it can be performed on young children who may be awake or lightly sedated to obtain multiple complete breathing cycles. The Rapid thoracoabdominal compression and Raised volume rapid thoracoabdominal compression uses devices that generate an external positive pressure to produce forced expiration, which can overlap with tidal breathing, resulting in a dynamic test like traditional spirometry. Tidal Breathing Flow-Volume Loop (TBFVL) parameters are used to classify different respiratory pathologies. These methods are a good choice for diagnosing and monitoring lung development and function. They can reveal the different outcomes of various respiratory diseases, even those resulting from respiratory infections (33).
**Diffusion Capacity of Lungs for Carbon Monoxide (DLCO)/Transfer Factor For CO (TLCO)**	The test is based on the affinity of carbon monoxide for hemoglobin and can be measured through an infrared detector or gas chromatography. It determines the lung’s ability to transfer oxygen to the body and is useful in restrictive respiratory pathology. It helps differentiate between pulmonary and extrapulmonary causes in children. However, the test’s low standardization for the respective correlation to the interpretation is a limiting factor (33).
**Lung Volumes (Body Plethysmography)**	Plethysmography collects all lung volumes as well as airway resistance, it is a valuable test to differentiate restrictive from obstructive pathology in children as well as monitoring lung function and respective response to therapy in the progression of a lung disease. This test is performed in a sealed chamber that is able to collect all the data of lung volumes and that is why there are some contraindications for its use such as claustrophobia, in addition to the use of certain devices that interfere with a basal respiratory exchange (33–36).
**Multiple-Breath Washout Test (Lung Clearance Index)**	This test evaluates the efficiency of an inert gas, such as nitrogen, in the respiratory process. Originally reserved for patients with cystic fibrosis, it has recently been shown to be useful in obstructive pathology. The lung clearance index (LCI) is a useful indicator for detecting early stages of peripheral airway pathology in small patients. It is measured with tidal breathing and reflects the complete respiratory cycles required to eliminate the inert gas (33–36).
**Exercise Testing -Cardiopulmonary Exercise Test – (CPET)**	CPET is a valuable test to determine the elicitation of abnormalities in respiratory function and exercise-induced asthma through a stressful stimulus such as controlled exercise, as it allows for the diagnosis, management and prognosis of pathology triggered solely by stressful situations. This test is performed in a controlled environment for aerobic exercise such as treadmill running and evaluates parameters such as heart rate, oxygen saturation, blood pressure, tidal volume, oxygen consumption rate, carbon dioxide elimination rate, etc. (33).

These tests are useful for early detection of pulmonary pathology and monitoring disease progression. However, it is important to note that many of these methods lack standardization and require reference values based on studies with similar populations and diagnostic teams.

## 5 Hormonal influences on lung morphogenesis and development

Mother’s hormonal environment has a great impact on lung development in the embryonic period (37). The impact of certain hormones, such as glucocorticoids, thyroid hormones, and insulin, on tissue development is widely recognized, and glucocorticoid therapy in preterm delivery is now part of the main guidelines as evidence of this. However, other hormones have also been shown to modulate the maturation of various organs, including the lung. These are hormones such as ghrelin, leptin, glucagon-like peptide 1 (GLP-1), retinoids and cholecalciferol. In the fetal lung, the leptin gene is expressed in lipofibroblasts and its levels increase during alveolar differentiation, when the synthesis of lung surfactant phospholipids is induced. Moreover, the lung is one of the few tissues expressing the Ob-R leptin receptor during fetal development (37). As for GLP-1, its receptor is expressed in lung tissue during fetal development, and its expression increases at the end of pregnancy, precisely when surfactant demand for alveolar expansion increases (38). Retinoic acid plays an essential role in cell differentiation and tissue maturation; low levels may cause fetal malformations, while excessively high levels may induce teratogenesis (39). During fetal lung development, retinoic acid regulates the formation of bronchial tubules, so a deficiency can cause pulmonary hypoplasia (40). Regarding vitamin D, severe 25(OH)D deficiency in premature infants has been shown to be related to respiratory distress syndrome, and its supplementation may reduce the time of assisted ventilation and may represent an effective means of preventing childhood asthma (41, 42). Another hormone with a relevant but still unclear role is ghrelin, which contributes to the distribution of energy resources during organ development. It has been shown that in small for gestational age (SGA) fetuses’ ghrelin levels are reduced, while there is an increase in cortisol due to the stress of the uterine environment (43).

To demonstrate the fundamental role that hormones have on lung development and function, Rosa et al. investigated the consequences of a disruption of the maternal hypothalamic-pituitary-adrenal axis during pregnancy on 222 mother-child pairs (44). An association was noted between reduced cortisol and increased toxins such as lead, which worsens lung function. This is certainly a complex interaction involving several factors, but it is an interesting point of study.

The exploratory research conducted by Prince et al. (45) examines the link between increased corticosteroid levels during the third trimester of pregnancy and the occurrence of infections in children. Prior studies have established the critical role of corticosteroids in fetal development, especially in the maturation of the lungs and immune system. This research seeks to determine if higher corticosteroid levels in the third trimester are associated with a lower frequency of infections in children. The findings revealed that elevated corticosteroid levels during this period were connected to a decreased incidence of infections in offspring. Notably, children whose mothers had higher corticosteroid levels experienced fewer respiratory and other common childhood infections compared to those with lower levels. The study accounted for various confounding factors, such as socioeconomic status, maternal health, and other prenatal conditions, to ensure the reliability of the results. The authors propose that the protective effect of elevated corticosteroid levels may stem from enhanced maturation of the fetal immune system. Corticosteroids are known to accelerate lung development and possibly enhance immune function, which could explain the reduced infection susceptibility in early childhood. Furthermore, the study underscores the significance of hormonal regulation during pregnancy and its lasting impact on child health.

Prince et al. conclude that higher corticosteroid levels in the third trimester are advantageous in lowering the risk of infections in children. This discovery has potential implications for prenatal care, suggesting that monitoring and potentially managing corticosteroid levels could improve health outcomes for offspring. Further research is suggested to explore the mechanisms behind this association and to assess the long-term effects of increased prenatal corticosteroid levels. The study paves the way for future research to investigate the specific pathways through which corticosteroids affect immune development. It also indicates the need for longitudinal studies to follow the health outcomes of children into adolescence and adulthood, considering prenatal corticosteroid exposure (45–47).

## 6 Hormonal influences on lung function

**Figure 2:**
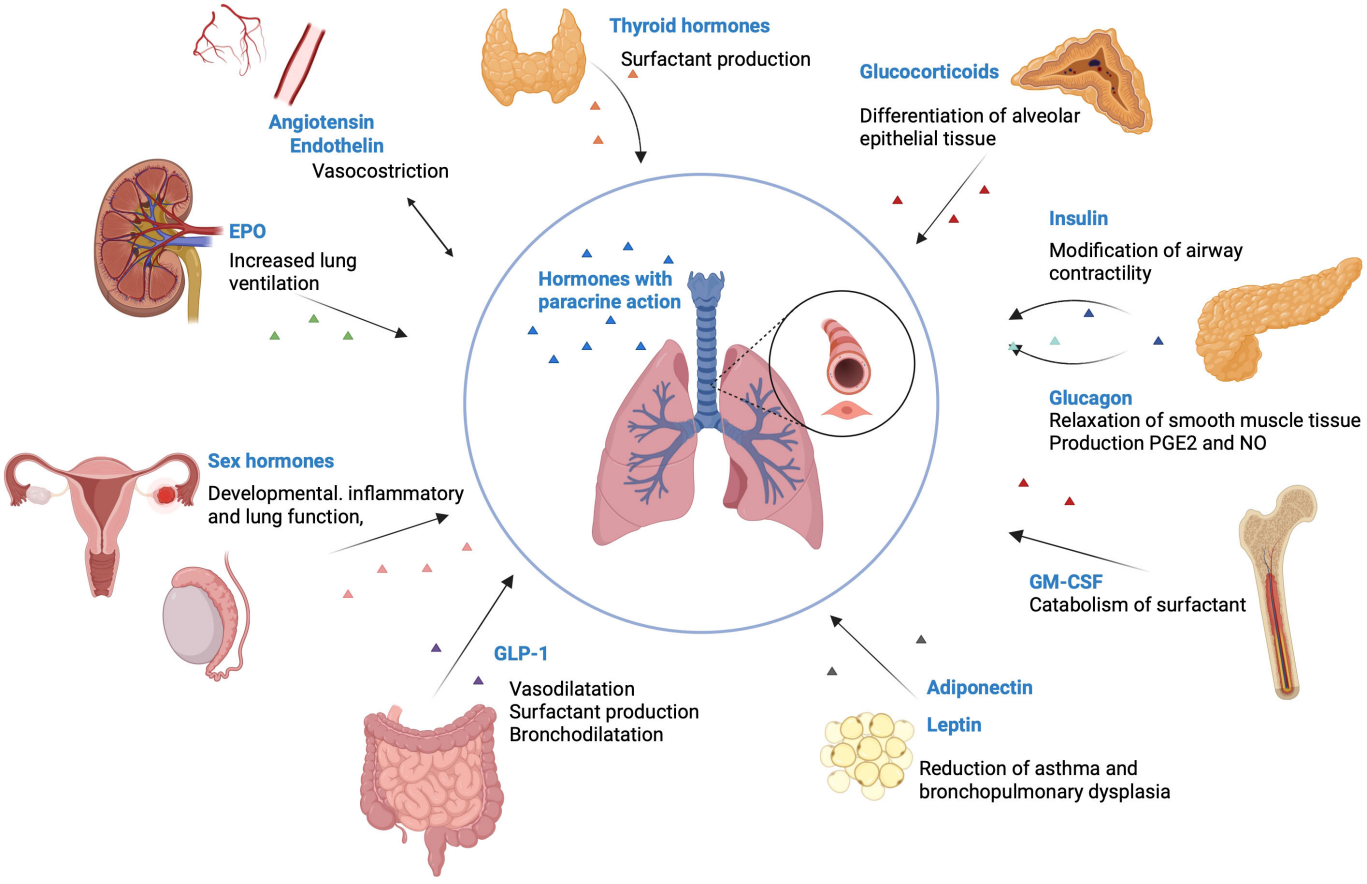
Hormones that influence pulmonary function. Thyroid hormones are involved in the production of surfactant. Glucocorticoids are essential for the differentiation of alveolar epithelial tissue. Insulin has an inverse association with the lung function, by modifying the airway contractility. Glucagon stimulates the relaxation of smooth muscle tissue and increases the production of PGE2 and NO. GM-CSF receptor increases the catabolism of surfactant. Adiponectin and leptin reduce the incidence of asthma and bronchopulmonary dysplasia. GLP-1 stimulates vasodilatation, surfactant production and bronchodilatation. Sex hormones influence developmental, inflammatory and lung function, by modifying NO levels, inhibiting airway epithelial secretion, and modifying cilia mechanics. Endothelin makes vasoconstriction in the lung parenchyma and in the airways. EPO increases lung ventilation through increased respiratory rate and tidal volume. Created with BioRender.com ^®^. EPO, Erythropoietin; GM-CSF, Granulocyte-macrophage colony-stimulating factor; GLP-1, glucagon-like peptide 1; PGE, prostaglandins; NO, Nitric oxide.

The endocrine mode of smooth muscle regulation in the airway is an aspect that has been largely overlooked compared to the paracrine mode of regulation (3). Smooth muscle contractility is influenced by hormonal factors produced by various organs, such as the pancreas, adrenal glands, adipose tissue, gonads, heart and gastrointestinal system, through hormones such as epinephrine, glucocorticoids, insulin, leptin, estrogen, progesterone and thyroid hormones (**Figure 2**).

### 6.1 Sex steroid hormones

It is widely known that sex differences are present in the incidence of pulmonary disease. For example, in the pediatric population, asthma is more common in males, while among adults, it predominantly affects females, and the reversal of this trend occurs during puberty. The female-male gap in asthma burden narrows around 50 years old. The sex reversal in asthma burden around major reproductive events suggests that sex hormones may play a role in the etiology of asthma (48). In chronic obstructive pulmonary disease (COPD) incidence, too, there are sex differences, since women - especially never-smokers - are more affected than men (5).

Sex hormones such as estrogen, progesterone and testosterone do not only play a role in the reproductive organs (10). Receptors for estrogen and progesterone have been found at the level of bronchial epithelial cells; several studies have shown the presence of these receptors also at the level of the lung parenchyma, as well as the presence of enzymes such as aromatase and steroid dehydrogenase, which proves the existence of local hormonal regulation (49).

Through their interactions with their widespread receptors, estrogens probably influence developmental, inflammatory, and disease processes in the lung. However evidence about their proinflammatory or antiinflammatory role is inconclusive (50). Some studies have witnessed a role for estrogen in chronic Th2-inflammation in the lung, participating in structural changes and leading to a worsening of lung function over time (51). Parity and polycystic ovarian syndrome (PCOS), which are related to an increase in circulating estrogen, have been identified as risk factors for inflammatory lung diseases including COPD (52). Estrogens may also contribute to the production of toxic metabolites in the airways of female smokers, participating in COPD pathogenesis (53).

Progesterone’s effects on the lung are far less studied, but they seem to include inducing relaxation of bronchial smooth muscle (49). Studies on androgens, on the other hand, have mainly shown a protective role in allergic airway inflammation, fulfilled by decreasing Th2 cell response (54). This might explain why in males asthma’s burden remains relatively unchanged from puberty until aging, whereas when testosterone starts reducing, an increase in asthma is observed (10). In Pesce et al. study, decreased DHEA-S levels in women have been associated with impaired lung function and a greater risk of developing airflow limitation later in adult life (55).

Influence of sex hormones on lung function is confirmed also by changes in organ’s functionality in different phases of the menstrual cycle. In Jeon et al. study on 103 teenage girls the forced expiratory volume (FEV) 1 was significantly lower during the menstruation period than during the rest of the time (56). Other studies have shown a decrease in FEV1 in women during the follicular phase, but no association during the luteal phase (57, 58). Mandhane et al. demonstrated that during natural menstrual cycles, eNO levels were negatively associated with estrogen levels and positively associated with progesterone levels, while these effects were not observed among women using oral contraceptives (59).

In postmenopausal women, an accelerated decline in lung function has been observed and it is likely attributed to the decreasing levels of estrogen in this phase (10, 60). Some authors have hypothesized that long-term oral hormone replacement therapy (HRT) could reduce loss of lung function and a recent study of Triebner et al. demonstrated a significant decreased loss of lung function over a 20-year period, consistent when adjusting for potential confounding factors (61). However, evidence of the effects of HRT is contradictory (52).

Studies show that women with cystic fibrosis (CF) have worse outcomes than men. Specifically, after puberty, females experience a higher number of pulmonary exacerbations associated with the disease compared to males (62). Holtrop et al. conducted a study on 23 women and found that during times of peak estrogen, there was a simultaneous increase in proinflammatory cytokines and a corresponding decrease in lung function (63). A possible explanation is that in CF, estradiol has been demonstrated to up-regulate mucin 5B (MUC5B) gene in human airway epithelial cells and inhibit chloride secretion in the airways (53).

Not all lung diseases have the same pattern of gender predominance as asthma, COPD and cystic fibrosis. Idiopatic Pulmonary Fibrosis (PF) is a restrictive lung condition characterized by lung inflammation and scarring, which leads to a progressive decline in forced vital capacity (FVC), FEV1, and total lung capacity (TLC). PF is more prevalent in men compared to women (64) and it tends to progress faster and results in lower survival rates in men (65). Interestingly, mortality rates in women are increasing at a faster rate than in men, suggesting that the current sex differences in PF may soon diminish or even reverse. Although our understanding of the biology of PF is growing, research on sex differences has mainly been conducted using animal models. The role of sex steroids in PF is still not well understood. Estrogens have been shown *in vitro* to increase the release of profibrotic factors in lung fibroblasts. One study indicated that airway fibrosis is influenced by both relaxin and estrogen, with estrogen providing a protective effect when relaxin is absent (66). There is a lack of *in vitro* data on the development and progression of PF, and many studies on the effects of sex steroids on fibroblasts and extracellular matrix proteins have not specifically focused on the lungs. Given the rapidly increasing mortality rates in women, it is important to thoroughly investigate the relationships between sex hormones and fibrosis (5). In addition, in contrast to the adverse effects of estrogens in chronic inflammatory lung disease, estrogens may be protective against airway fibrosis while androgens may be harmful (67).

In non-cystic fibrosis bronchiectasis, studies suggest that progesterone may impair cilia beat frequency in airway epithelium, reducing the ability to expectorate and leading to an increase in bacterial load and likelihood of exacerbations (68).

Lung disease due to non-tuberculous mycobacteria (NTM) is disproportionately increased in postmenopausal women, suggesting estrogen deficiency and altered adipokine values might take part in its pathogenesis (69). Another valid demonstration in support of this thesis is the increased incidence of NTM pneumonia in patients with hypopituitarism.

Sex steroid hormones play also a significant role in lung repair, influencing both the immune response and tissue regeneration processes. Specifically, research has shown that progesterone has protective effects on the lungs, especially during infections like influenza. Progesterone helps with lung repair by reducing inflammation and increasing the production of amphiregulin, a growth factor that assists in tissue repair. Experimental studies have demonstrated that progesterone can reduce lung damage caused by the influenza A virus by modulating the immune response and promoting tissue repair.

Additionally, estrogen has been shown to reduce inflammation and assist in the repair of lung tissue, which could explain why pre-menopausal women often experience different disease progressions compared to men. Testosterone also affects lung function, and some studies suggest it has beneficial effects on lung capacity and repair mechanisms (70, 71) These data suggest that potential therapeutic applications for sex steroid hormones could be considered in lung diseases and injuries (70).

Several studies have also explored sex differences in the mechanisms of cancer development and progression, but comprehensive research in this area is still limited. One study on lung adenocarcinoma in a Chinese cohort found that tumors in men have a higher burden of genetic alterations, which is associated with poorer overall survival rates. Univariate analysis also revealed a connection between sex and smoking status, with men and smokers showing a higher mutation burden (72). Other findings showed higher levels of polycyclic aromatic hydrocarbons (PAH)-DNA adducts in women, which are used to assess the potential for lung cancer development. Hormonal regulation was suggested as a possible factor, based on previous evidence of interactions between estrogen receptors and aryl hydrocarbon receptor signaling pathways, which are believed to influence PAH-metabolizing enzymes (73). Other proposed mechanisms in cancer development, such as differences in DNA repair capacity and the induction of cell proliferation by bombesin-like peptides, have limited data on sex differences. The only study on DNA repair capacity found that women have a lower DNA repair capacity than men. Another mechanism involved in cancer development is nicotine exposure, which promotes cellular proliferation, tumor growth, migration, and invasion (74). Interestingly, female sex and the use of oral contraceptives have been shown to alter nicotine metabolism, leading to higher levels of toxic nicotine metabolites. Early *in vitro* studies showed that female airway cell cultures exposed to cigarette smoke produce more oxidants, potentially causing airway damage and the development of lung diseases such as cancer (75, 76).

### 6.2 Leptin and adiponectin

The role of hormones produced by adipose tissue has been widely studied in the context of asthma. In Yuksel et al. study about 111 children, asthma was more severe in the obese children with obesity with asthma than in the children without obesity and in the second group leptin and adiponectin levels were correlated with the asthma symptom score (77). In particular, several studies found higher levels of leptin and lower levels of adiponectin in association with asthma and a correlation between these hormonal alterations and the severity of respiratory disease, both in adults and in children (78, 79).

One increasingly interesting aspect is the impact of these hormones on lung development during intrauterine life. Blanche et al. in an original study from 2021 analyzed the relationship between leptin and adiponectin and pediatric lung function, specifically assessing the increased risk of developing asthma (80). Correlating adipocytokine concentrations in umbilical cord blood and respiratory clinic and spirometry of the same children up to 5 years of age, it was found that higher concentrations of adiponectin in cord blood were associated with a higher FEV1 in females, but not in males. Higher leptin was associated with a lower risk of developing asthma in females, but not in males, while higher concentrations of adiponectin were associated with reduced asthma in both males and females. Furthermore, those born preterm or small for gestational age, who have low concentrations of adiponectin, have a higher risk of developing bronchopulmonary dysplasia due to oxidative stress and inflammation (81).

### 6.3 Insulin

The evidence that insulin resistance, a complication in which insulin is increased, is common in children with asthma and is associated with asthma-like symptoms in adults, has suggested an inverse association between blood levels of insulin and lung function (3, 82–84). Among the various mechanisms by which insulin may alter the development or severity of asthma, its adverse effect on airway contractility has been widely reported (3).

A single center cross-sectional study was conducted in Turkey in 2015, the aim of which was to determine the role of insulin resistance in lung function in a group of patients admitted to internal medicine. The results of the study showed a greater decline in lung function in the group of patients with insulin resistance, demonstrating that insulin resistance is a factor that contributes to the decline in lung function (85).

Another study conducted in 2016 attempted to fill the gaps in the existing knowledge regarding the relationship between hyperinsulinemia and lung function. In particular, insulin stimulates the production of smooth muscle cells, collagen production and the activation of beta-catenin: these elements make hyperinsulinemia one of the factors triggering the hypercontractility responsible for the asthmatic phenotype (86).

The metabolic condition of insulin resistance and insulin insufficiency also plays a role in the disruption of collagen and elastin cross-linking, leading to decreased lung elasticity, which influences the risk of pulmonary disorders, particularly in patients with diabetes.

An increased prevalence of pulmonary disease is observed in type 1 and type 2 diabetes patients (87, 88) including idiopathic pulmonary fibrosis, COPD, asthma, and lung cancer. Diabetes is a leading cause of comorbidity in several lung pathologies. Although several mechanisms have been suggested, mainly associated with the pro-inflammatory and proliferative properties, as well as the micro- and macrovascular effects of diabetes, a direct effect of insulin on structural cells and immune cells in the airway has also been proposed as a link between diabetes and lung diseases (88). This is further confirmed by the observation that metformin is considered a potential therapeutic agent in lung diseases, while insulin has been shown to exacerbate lung diseases (88).

### 6.4 GM-CSF

As reported by Rangel et al. (4), lung function is influenced by cytokines and hormones, including GM-CSF. Specifically, alveolar macrophages and type II pneumocytes express the GM-CSF receptor, which increases the catabolism of surfactant (72). Therefore, a potential mechanism linking increased GM-CSF levels with alveolar collapse and decreased airway patency is that GM-CSF reduces the amount of alveolar surfactant (through its accelerated catabolism), thereby increasing surface tension and lung elastance. Furthermore, high surface tension not only promotes alveolar collapse but also small airway closure (73).

### 6.5 Glucagon

Glucagon stimulates the relaxation of smooth muscle tissue in various organs, including the esophagus, intestine, vascular system, and airways, not only by stimulating smooth muscle glucagon receptors, but also by increasing the production of Prostaglandin E2 (PGE2) and nitric oxide (NO) at the epithelial level (89). Glucagon receptors are GAS protein-coupled receptors, which therefore lead to an increase in intracellular cAMP; the same signaling pathway is exploited by b2-adrenergic drugs, the most widely used class of bronchodilator drugs. The functional closure of the airways is the factor that determines the limitation of forced exhalation, and therefore determines the FVC; the airways are kept open by pulmonary surfactant and transpulmonary pressure forces, which depend on the state of contractility/relaxation of smooth muscle cells. Glucagon, by reducing the contractility of pulmonary interstitial cells, could reduce the pressure forces that favor the increase in FVC (90).

### 6.6 GLP-1

According to some recent studies, the GLP-1 may have some impact on lung function, too (91, 92). GLP-1 receptor is largely distributed in the lung and, through its receptor, GLP-1 stimulates vasodilation, surfactant production, and bronchodilation. Although there is no evidence of GLP-1 production within the lung, according to Mendivil et al. study its concentrations are higher within bronchoalveolar fluid as compared to the serum (93). It has been hypothesized that decrease in GLP-1 production with obesity and diabetes may contribute to lung dysfunction. A potential explanation is that in obese patients inflammation due to interaction between advanced glycation end-products (AGE) and their receptor (RAGE) in the lung potentiates asthma, and GLP-1’s role in attenuating this mechanism, reducing RAGE expression and downstreaming their signaling, is decreased because of its reduction.

### 6.7 Erythropoietin, endothelin and angiotensin

Rubini et al. analyzes the role of factors such as angiotensin, endothelin and erythropoietin on the respiratory mechanics, and in particular on the visco-elastic pressure components of beginning and end-expiration (94). The exact mechanisms are still partly to be elucidated, but it is clear that several biochemical factors can influence the mechanical interactions between elastin, collagen and interstitial fluids. According to Rubini et al., erythropoietin appears to increase lung ventilation through increased respiratory rate and tidal volume. It also reduces airway resistance through receptors in smooth muscle.

Endothelin is an endothelial factor that induces vasoconstriction at the level of blood vessels, and it has been shown to induce this same effect at the level of the airways and at the level of the lung parenchyma (95).. Magalhaes et al. studied the link between plasma angiotensin levels and bronchoconstriction in asthmatic subjects (96). It would appear that angiotensin also plays a role in the respiratory physiology of healthy subjects.

### 6.8 Growth hormone

The lung is well established as a postnatal target site for growth hormone (GH) action, given that pathophysiological states of GH excess and deficiency are both associated with impaired pulmonary function (97). In conditions of GH excess, such as acromegaly, respiratory disorders are a common complication: patients often present with structural and functional lung changes, including tracheobronchomegaly, airway abnormalities and ventilation inhomogeneity. These conditions indicate that elevated levels of GH may disrupt the normal architecture and function of the lungs, although the precise correlation between GH levels and these changes remains unclear (98, 99).

Conversely, in cases of growth hormone deficiency (GHD), children exhibit impaired lung function that is not solely attributable to reduced body size. Rather, this impairment is also a consequence of the direct effect of GH on the lungs (100). Treatment with recombinant human growth hormone (rhGH) has been shown to improve some pulmonary parameters, although results are inconsistent, particularly in populations with underlying conditions like cystic fibrosis (CF). In children with CF, rhGH therapy has shown some promise in improving growth parameters like height, weight, and lean body mass, although consistent benefits in lung function are not uniformly observed across studies (101).

It has been proposed that the lung may itself be a site of GH production during prenatal development. Some studies have identified G and its receptors in the lungs from early developmental stages, before systemic endocrine regulation is established, indicating the potential for autocrine or paracrine functions of GH in the organ. This is demonstrated by the expression of GH-related genes in the lungs of both chicks and rats during embryonic development (97, 102).

The aforementioned evidence serves underscore the significance of GH in respiratory health and the necessity for a more comprehensive understanding of the hormonal influences on the lungs, with a view to optimizing therapeutic approaches.

### 6.9 Thyroid hormones

Thyroid hormones play a role in the development of the fetal lung, although it is not well-defined. Triiodothyronine (T3) and thyroxine (T4) are involved in the production of surfactant phospholipids, particularly phosphatidylcholine, by binding to specific receptors in the pulmonary cells. Furthermore, thyroid hormones have been observed to reduce the activity of protective antioxidant enzymes in the developing lung (103).

The influence of thyroid hormones on lung function persists beyond the perinatal period. The presence of thyroid hormone receptors in lung tissue provides evidence for a direct role of these hormones in modulating respiratory system functions. Hypothyroidism may result in the weakening of respiratory muscles and a reduction in lung capacity, whereas hyperthyroidism may lead to an increase in airway resistance and a decrease in lung volume. Furthermore, an imbalance in thyroid hormones has been associated with an array of respiratory disorders, including COPD, asthma, lung fibrosis, and sleep-disordered breathing (104, 105).

Thyroid hormones aid also in sustaining cellular integrity and function under these stressful conditions, reducing inflammation and supporting cell survival and repair (71).

### 6.10 Epinephrine

In the lungs, epinephrine exerts its effects primarily through the activation of beta-adrenergic receptors, which lead to bronchodilation. Epinephrine’s ability to relax bronchial smooth muscle and reduce airway resistance makes it a critical therapeutic agent in emergency respiratory management.

Furthermore, it affects the rate and depth of respiration, thereby enhancing oxygen intake during stress responses (106).

### 6.11 Glucocorticoids

Glucocorticoids play an essential role during fetal lung development, as they are essential for the differentiation of alveolar epithelial tissue. Prenatal administration of glucocorticoids is in the guidelines for the prevention of neonatal respiratory distress syndrome, because it promotes lung maturation.

Several maturation processes occur prenatally, including the production of surfactant needed to reduce alveolar surface tension. The glucocorticoid receptor is expressed in the fetal lung and stimulates the production of surfactant-associated proteins, increasing phosphatidylcholine activity. Glucocorticoids also stimulate cellular maturation and differentiation, inhibition of DNA synthesis, interstitial tissue composition, production of antioxidant enzymes, and regulation of lung fluid metabolism, even postnatally (107).

The most commonly used anti-inflammatory drugs for treating airway diseases are topical glucocorticoids, which act by suppressing inflammation and preventing the recruitment of inflammatory cells. Glucocorticoids act through specific nuclear receptors, and as such have a role in modulating gene expression. Target genes include inflammatory mediators such as chemokines, cytokines, growth factors, and others (108).

Glucocorticoids suppress inflammatory genes that are activated in asthma by reversing the acetylation of histones of inflammatory genes. At higher glucocorticoid concentrations, GR homodimers interact with DNA recognition sites to activate transcription through increased histone acetylation of anti-inflammatory genes and transcription of several genes related to glucocorticoid side effects (trans-activation). Glucocorticoids also have post-transcriptional effects and reduce the stability of some proinflammatory mRNAs. Patients with COPD or severe asthma have reduced responsiveness to glucocorticoids, as oxidative stress resulting from severe inflammation greatly reduces the phosphorylation activity and post-translational modifications of GCs (109).

### 6.12 Melatonin

Currently, studies on the role of melatonin in lung function are almost exclusively in laboratory animals. Early results are showing that melatonin significantly reduces mortality and restores alveolar epithelial function, which is linked to mitochondrial integrity. Melatonin appears to reduce ROS production, prevent apoptosis and senescence of type II alveolar cells, and improve mitochondrial dysfunction through regulation of apelin 13 (110).

New evidence suggests that melatonin promotes antioxidant actions in contexts such as sepsis and cardiopulmonary diseases, during which the differentiation processes of alveolar epithelial cells are disturbed. Ning et al. (111) suggests that melatonin could have a protective role in sepsis-induced acute lung injury (ALI). By gene knock-out of melatonin receptors at the alveolar level, the study demonstrated how pretreatment with melatonin significantly inhibited pathological damage, inflammatory response, oxidative stress and apoptosis in lung tissues, inhibiting fatty acid oxidation in lung epithelial cells and acting on mitochondria, in particular on the SIRT3 gene. These data could be very useful in the future, to exploit melatonin as a treatment for ALI.

The implication of melatonin in lung function is also found in the context of nocturnal asthma: up to 40% of asthmatic subjects are awakened by asthma every night, due to circadian variations in airway inflammation and increased smooth muscle hyperreactivity, a phenomenon in which glucocorticoids and melatonin participate (112).

### 6.13 Oxytocin

The few studies conducted to date on oxytocin have been performed on experimental. It has been reported that oxytocin is effective in animal models for treating acute lung injury, reducing the inflammatory pathways of Toll like receptor -4 (TLR4) e NOD-like receptor protein 3 (NLRP3) (113).

In addition to acute lung injury, oxytocin also appears to be beneficial in patients with obstructive sleep apnea syndrome (OSAS). An interesting study was conducted in 2017, which tested nocturnal oxytocin administration in eight patients with OSAS, assessing changes during sleep in apnea, hypopnea, heart rate, and arousals. Oxytocin significantly increased indices of parasympathetic activity, including heart rate variability, total sleep time, and the post-polysomnography sleep assessment score, an index of self-reported sleep satisfaction (114). It did not reduce apnea events, but significantly reduced hypopnea events. Further studies are needed to better understand the mechanisms by which oxytocin promotes these changes in cardiorespiratory and autonomic function in patients with OSA; probably there’s a link with upper airway muscle tone and reactivity, predisposing to greater or lesser laxity.

The oxytocin receptor is ubiquitous, so it is now clear that its role is not only linked to pregnancy, milk ejection and uterine contractions, but that it regulates inflammatory responses in general. We cite a study conducted in 2010, whose aim was to define the role of oxytocin in the modulation of airway smooth muscle, in the presence and absence of interleukin (IL)-3 and tumor necrosis factor (TNF)-α, pro-inflammatory cytokines that play an important role in pathological mechanisms such as asthma (115). The expression of the oxytocin receptor is strongly influenced by the presence of these cytokines; in addition, oxytocin stimulates the increase of cytosolic calcium within the cells of the airway smooth muscle.

## 7 Sex difference in respiratory physiology

Sex-disparities may manifest across a spectrum of parameters, including lung dimensions, capacities, mechanics, and responsiveness to external stimuli. Differences emerge already during prenatal development (16-24 gestational weeks) and persist throughout one’s lifespan (116). Female fetus manifests an earlier lung maturation and surfactant production which helps keep small airways open, compared to males. Additionally, maternal and/or fetal sex steroids may influence lung development (101–103). These inherent and steroid-induced sex differences in lung development are clinically relevant, particularly in understanding the increased susceptibility of premature male infants to conditions such as respiratory distress syndrome (RDS) and bronchopulmonary dysplasia (BPD) (101–103). Moreover, disparities in lung structure and function established early in life may impact the trajectory of diseases like asthma in both childhood and adulthood. Postnatal lung development primarily involves exponential increases in the number and size of alveoli. At birth, the female lung is smaller than the male lung and has fewer respiratory bronchioles, but the number of alveoli per unit area remains comparable. Consequently, males have a greater total number of alveoli and surface area throughout childhood (7, 116, 117). Women generally possess smaller lung dimensions, evident across all respiratory system levels from upper airways to lungs, persisting from fetal development through adulthood. During childhood and adolescence, dysanapsis occurs, indicating uneven organ growth while maintaining overall physiological functionality. Specifically, males experience delayed airway development compared to lung growth, unlike females airways and lung parenchyma grow proportionally. Consequently, males may have a disproportionately lower alveolar count relative to airway numbers, resulting in reduced expiratory flow rates even after reaching full physical maturity. Adolescent males generate increased respiratory pressures due to testosterone (T) influence and alterations in thoracic shape, respiratory muscle strength, and dynamics, affecting breathing patterns and respiratory efficiency. The increase in FVC with age is indeed more marked in men in their early postpubertal years, partly due to the higher airway pressures that men produce as a result of the male sex hormone effect (5, 7, 116, 118). Emerging evidence suggests that sex hormones exert influence on airway dynamics and inflammatory processes within the pulmonary system. The impact of estrogens (E2) on pulmonary function and pathology remains contentious, likely due to the complex expression of estrogen receptors across various cell types and the diverse effects of estrogen. Studies indicate E2 may modulate the progression of developmental, inflammatory, and pathological pathways by upregulating and concurrently inhibiting cytokines and inflammatory mediators. Furthermore, investigations suggest that E2 is associated with heightened respiratory drive, potentially resulting in increased minute ventilation and respiratory rates in females compared to males. Additionally, it may enhance respiratory muscle function, thereby contributing to improved ventilatory efficiency and respiratory performance (119). Indeed, progesterone (P) appears to influence airway tone by reducing bronchial smooth muscle contractility and promoting relaxation, possibly leading to bronchodilation (7). In humans, P levels are positively correlated with peak expiratory flow rate during the luteal phase of the menstrual cycle. T, the primary male sex hormone, is also present in females, albeit at lower concentrations. Notably, T exhibits protective properties by inducing bronchial tissue relaxation, reducing responsiveness to histamine, and mitigating airway inflammation through its influence on cytokines and inflammatory mediators within the lung. Studies have demonstrated a correlation between T levels and respiratory muscle strength, with higher levels associated with increased muscle mass and force generation (7, 120).

In the realm of literature, there exists a significant divergence of opinions concerning the potential gender disparities in ventilation dynamics (**Table 2**). Certain investigations revealed elevated ventilation (VE) rates among men, particularly during specific phases of the menstrual cycle, while others have documented higher VE among women under normal physiological conditions (2, 121). Conversely MacNutt et al. observed a notably lower absolute VE rate in women compared to men, although this discrepancy lessened upon adjustment for body surface area (122). Recent research endeavors, characterized by extensive participant cohorts, have confirmed that women generally exhibit a higher baseline respiratory rate, but lower baseline VE compared to men, even after correcting for differences in body surface area (123). The inconsistency in findings is partly attributed to challenges in assembling homogeneous subject cohorts. A multitude of studies have elucidated the role of E2 in amplifying the effects of P on VE. However, combined administration of E2 and P augmented VE and VT)during normal respiration without affecting respiratory rate. Some investigations have indicated that elevated levels of P and its receptors are imperative for stimulating VE. Nonetheless, it’s crucial to note that VE remained unaffected when a physiological dosage of E2 and P was administered (2). Additionally, heightened levels of circulating T have been shown to influence respiratory parameters in males. Various studies have demonstrated that high doses of T increase minute ventilation, although the magnitude of this effect depends on the dosage administered; supraphysiological doses augmented VE, whereas lower doses exhibited no effect or even reduced VE (124, 125). Sex responses to hypoxia and hypercapnia are different and research shows conflicting viewpoints. Numerous investigations have examined the disparity in hypoxic ventilatory chemosensitivity between genders; nevertheless, the findings are equivocal. Studies in humans have indicated that the hypoxic ventilatory response (HVR) may be lower in females compared to males, equivalent across genders, or even higher in females (2, 123, 126). Concerning E2, studies conducted in human subjects indicated that supplementation with E2 alone does not induce alterations in HVR. However, when E2 is supplemented concurrently with P, there is a notable increase observed in HVR attributing this phenomenon to the induction or upregulation of P receptors by E2, as elucidated previously. Notably, P enhances activity within specific brain regions subsequent to E2 pre-treatment (127). In females, supplementation with both P and E2 has been demonstrated to amplify the ventilatory response to hypoxia when hormone replacement involves supra-physiological doses, potentially influencing ventilatory efficiency through non-specific mechanisms (2). Regarding androgens, findings from studies conducted in human subjects present contrasting results. While one study observed a decrease, another noted an increase in HVR subsequent to testosterone treatment in hypogonadal males (2). Data concerning the impact of sex on Hypercapnic Ventilatory Response (HcVR) are inconclusive. Some studies propose that males exhibit a higher ventilatory response to CO2 compared to females, while others report no significant disparity between the genders (123, 124). Studies conducted in human subjects suggest that postmenopausal females exhibit diminished central chemoreflex drive associated with decreased sex hormone concentrations, while treatment with specific hormones such as P may heighten ventilatory responses to hypercapnia (128). Thus, in women different phases of the menstrual cycle are associated with different ventilatory patterns likely due to estrogen and progesterone fluctuations on respiratory drive possibly affecting lung function parameters and airway sensitivity. In rest conditions, most studies report that respiration is higher in the luteal phase (LP), in terms of higher VE and VE/CO2 production, particularly with increasing sex hormones. These findings suggest a beneficial influence of female sex hormones on thoracic pump muscle strength during the LP. However, few studies report no significant differences in VE of LP versus follicular phase (FP) under baseline conditions (7, 122, 129).

The importance of these hormonal, structural and functional differences is also reflected in the increased susceptibility to the development of respiratory diseases. Take, for instance, asthma, which manifests more frequently and severely in young males, yet exhibits greater prevalence in adult females, who frequently experience perimenstrual asthma (PMA)—a worsening of respiratory symptoms during menstruation (116, 129). COPD may also manifest differently between males and females, showcasing variations in symptoms, lung function deterioration, and response to treatment. These distinctions could contribute to differences in symptom severity, treatment effectiveness, and disease progression (5). Sex hormones may directly influence respiration by impacting its primary control mechanisms, yet the precise role of each hormone in ventilatory control remains ambiguous. Despite considerable research endeavors, a comprehensive comprehension of how sex and sex hormones affect respiratory regulation remains elusive, with available data often inconclusive.

**Table 2:** List of studies showing the influence of sex on ventilation (VE) - adapted from (2).

Author	Population	Methods	Results
In normoxic normocapnic conditions			
Romei et al. (121)	Humans	plethysmography in quiet brathing	VE higher in men than in women
Goldberg et al. (123)	Humans	Re-breathing system	VE higher in men than in women
MacNutt et al. (122)	Humans	VE measured by pneumotachography	VE equal in men and women
In hypoxic conditions			
Caravita et al. (126)	Humans	VE measured by pneumotachography	HVR higher in men than in women
Goldberg et al. (123)	Humans	VE measured by pneumotachography. Hypoxic testing when SpO2 reached 75%	HVR higher in men than in women
In hypercapnic conditions			
MacNutt et al. (122)	Humans	VE measured by pneumotachography	HcVR of men higher than women
Goldberg et al. (123)	Humans	VE was measured by pneumotachography	HcVR equal in men and women when corrected by body mass

## 8 Limitations

This review has several limitations. First, it is a narrative review, which, as Gregory et al. (130) point out, provides a non-systematic summary and analysis of the literature on a specific subject. The lack of systematic methodology in narrative reviews can lead to potential biases in the selection of studies, and they typically result in qualitative rather than quantitative syntheses. Our search included publications from the past 20 years; expanding the research to cover the past 40-50 years could be useful to include other informative studies not analyzed in more recent manuscripts.

Secondly, we aimed to explore the effects of hormones on lung function, highlighting the importance of this understanding in developing personalized monitoring strategies in both medical and surgical pediatric settings. We have not fully analyzed the pharmacological impact of hormonal therapies, especially on patients with coexisting metabolic disorders and lung diseases, as it was not the primary focus of this work. However, this aspect will be considered in a future study due to its significance, particularly in terms of disease prognosis.

Additionally, most of the clinical data reported in the literature are based on results from male cohorts, and data for female cohorts are insufficient or missing. Further studies are necessary for a better understanding of sex disparities in the hormonal effects on lung development and function.

Finally, for some hormones, such as oxytocin, the reported studies have been predominantly conducted on animals. The effects of these hormones on humans need to be further investigated to be better understood.

## 9 Conclusions

Respiration is a vital physiological process that is controlled and regulated by a great many factors, allowing the preservation of adequate oxygen supply and acid-base balance in living tissues. In recent years, it has become apparent through more and more research how these mechanisms are not only related to muscles and hypoxemia/hypercapnia stimuli, but are instead finely regulated by hormonal signaling. In fact, the endocrine mode of regulation on airway smooth muscle is an aspect that has been largely overlooked compared to the paracrine model.

Hormones play an important role in the growth and development of lung tissues, influencing them from early stages through infancy, childhood, adolescence, and into adulthood. They have a broad impact, from shaping respiratory drive and muscle performance to modulating airway reactivity and lung maturation. From morphogenesis, in the embryonic period, the mother’s hormonal environment contributes to lung development: glucocorticoids, thyroid hormones, and insulin are critical for tissue development, knowledge now known to have allowed glucocorticoids to be used as therapy for lung development in fetuses at risk of preterm delivery, but other hormonal factors such as ghrelin, leptin, GLP-1, retinoids, and cholecalciferol are also critical for lung development. Sex steroids, hormones derived from adipose tissue, and factors like insulin, GM-CSF, and glucagon are key players in modulating respiratory mechanics and inflammation, central to shaping respiratory health. Specifically, the modulation of smooth muscle contractility is a crucial aspect in the intricate relationship between sex hormones and the incidence of lung disease and sex disparities.

While ample evidence underscores the impact of hormones on lung development and function, along with sex-related differences in the prevalence of respiratory disorders, further research is needed to clarify their specific roles in these conditions. Discrepancies in findings may be attributed to factors such as species variations, experimental methodologies, hormone dosages, and the menstrual cycle phase in female subjects—factors frequently overlooked in studies.

Considering the role of hormones in the structural and functional components of the lung is useful for better understanding the pathogenesis of pulmonary diseases at various stages of a child’s life. During the developmental stages, a child’s endocrinological profile differs from that of an adult. Integrating knowledge of hormonal influences on lung function, taking into account the specificities of pediatrics, into clinical practice can improve the accuracy and effectiveness of respiratory care interventions. A thorough understanding of the intricate network of physiological mechanisms that together contribute to normal lung function could enable the emergence of new therapies in the field of personalized medicine: by isolating the individual factors responsible for the physiological pathway, it might be possible in the future to modify and manipulate them in order to increase or conversely inhibit certain pathways, and consequently increase or inhibit a cascade of downstream effects.

Understanding the impact of hormones on lung function could be valuable for developing personalized monitoring approaches in both medical and surgical pediatric settings, in order to improve outcomes and the quality of care for pediatric patients.

Considering pulmonary pathology with a multidisciplinary approach tailored to pediatric age is useful for improving outcomes and the quality of care for children with pulmonary diseases.
